# Functional impact of splice isoform diversity in individual cells

**DOI:** 10.1042/BST20160103

**Published:** 2016-08-15

**Authors:** Karen Yap, Eugene V. Makeyev

**Affiliations:** * MRC Centre for Developmental Neurobiology, King's College London, London SE1 1UL, U.K.; † School of Biological Sciences, Nanyang Technological University, Singapore 637551, Singapore

**Keywords:** alternative splicing, isoform co-expression, nervous system development and function, single-cell analyses

## Abstract

Alternative pre-mRNA splicing provides an effective means for expanding coding capacity of eukaryotic genomes. Recent studies suggest that co-expression of different splice isoforms may increase diversity of RNAs and proteins at a single-cell level. A pertinent question in the field is whether such co-expression is biologically meaningful or, rather, represents insufficiently stringent splicing regulation. Here we argue that isoform co-expression may produce functional outcomes that are difficult and sometimes impossible to achieve using other regulation strategies. Far from being a ‘splicing noise’, co-expression is often established through co-ordinated activity of specific *cis*-elements and *trans*-acting factors. Further work in this area may uncover new biological functions of alternative splicing (AS) and generate important insights into mechanisms allowing different cell types to attain their unique molecular identities.

## Introduction

Alternative pre-mRNA splicing (AS) allows a single gene to generate more than one mature mRNA species through non-uniform utilization of exonic and intronic sequences [1]. Many multiexon transcripts in higher eukaryotes undergo AS. It is currently thought that this form of regulation effectively quadruples the number of protein isoforms compared with the number of their encoding genes in mammalian genomes [2–4].

AS occasionally functions as a tightly controlled switch that consistently generates one or another mRNA isoform depending on the circumstances. Such regulation is used, for example, during sex determination in *Drosophila*, where distinct splice isoforms of critical RNA-binding proteins and transcription factors are expressed strictly in a sex-specific manner [5]. Distinct AS isoforms can be also expressed in different tissues of the same organism providing an efficient means to adjust protein functions to local physiological requirements [6]. Implicitly, switching between AS patterns increases the number of mRNAs and proteins at the level of organism or population but not at the level of individual cells.

However, AS does not always follow this binary logic. Numerous examples have been reported over the years where AS isoforms are co-expressed at readily detectable levels in specific tissues and cell types [7–11]. Balanced production of different isoforms is often functionally important under normal conditions and deregulated in disease [12,13]. Yet tissues consist of different cell types, and even morphologically homogeneous cultures may contain physiologically and epigenetically distinct cells. Hence detecting co-expression in samples pooled from multiple cells does not guarantee that AS isoforms co-occur at a single-cell level. Some pre-mRNAs may be spliced in individual cells in a switch-like manner and analysing cells in aggregate could lead to misinterpreting this underlying bimodality as co-expression [14].

Nonetheless, as sensitivity and resolution of single-cell RNA detection technologies improve, it is becoming increasingly clear that isoforms can coexist in the same cell at physiologically relevant levels. In single-cell RNA sequencing (scRNA-seq) analyses, co-expression is often seen for transcripts expressed at relatively high levels and less affected by biological and technical noise than their low-abundance counterparts [15,16]. Several studies using single-cell RT-(q)PCR, single-molecule RNA FISH and other approaches potentially affording better sensitivity than current scRNA-seq protocols have also reported co-occurrence of splice isoforms in the same cell [17–22].

Does co-isoform co-expression serve a biological purpose or is it merely a result of loosely controlled AS? Here we discuss several examples where AS-mediated diversification of transcriptomes and proteomes of individual cells leads to functionally important outcomes. Focusing on the nervous system, where the impact of AS has been investigated especially well [23–25], we argue that isoform co-expression provides an efficient mechanism for generating self-recognition codes in neurons, modulating protein functions and maintaining gene expression homoeostasis.

## Generating self-recognition codes

Assembly of neuronal circuits is essential for brain development and function. This in turn requires a neuron to respond to chemical guidance cues, form selective synaptic connections and, importantly, distinguish its own neurites from those of other neurons [26]. Sister projections outgrowing from the same soma tend to avoid each other, and in both invertebrates and vertebrates, this behaviour relies on stochastic co-expression of distinct isoforms of surface proteins capable of highly specific homophilic interactions.

**Figure 1:**
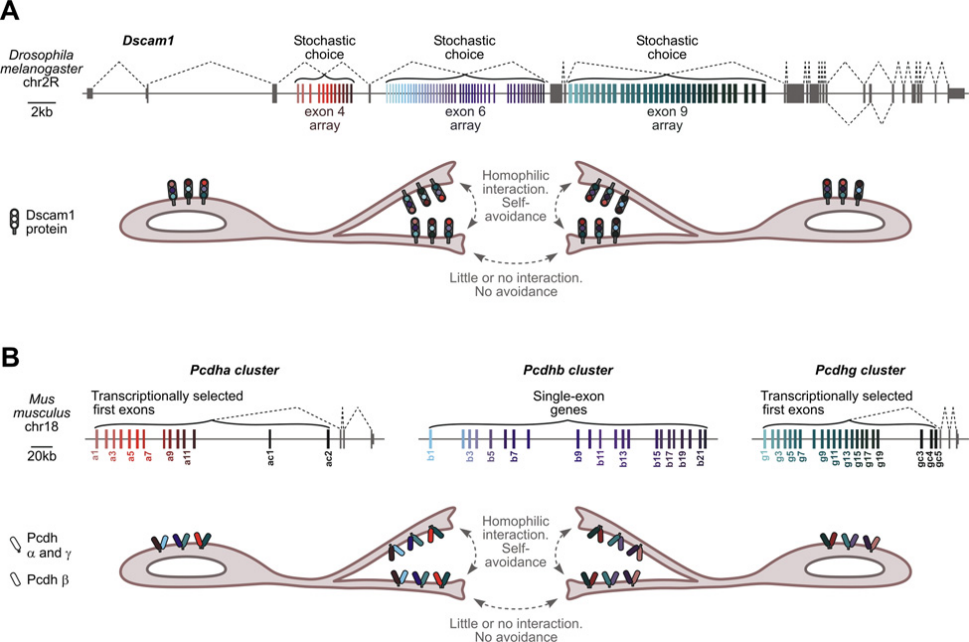
Role of isoform co-expression in generating self-recognition codes (**A**) *Top*, fruit fly *Dscam1* gene. The three alternative exon clusters are shown in colour. Other features, including the mutually exclusive pair of exons 17.1 and 17.2 are grey. *Bottom*, branches emanating from the same neuron express identical repertoires of Dscam1 isoforms. This promotes homophilic interaction between Dscam1 ectodomains *in trans* and ultimately turns on the self-avoidance programme [26]. Branches from different neurons express distinct Dscam1 repertoires and therefore do not interfere with each other. (**B**) *Top*, mouse protocadherin clusters. Variable first exons in the *Pcdha* and the *Pcdhg* clusters as well as entire single-exon genes in the *Pcdhb* cluster are shown in colour. Constitutive *Pcdha* and *Pcdhg* exons are grey. *Bottom*, protocadherins promote dendritic self-avoidance in a manner largely similar to *Drosophila* Dscam1. An important nuance is that, in addition to homophilic interactions *in trans*, Pcdhs can form heterodimers and possibly higher-order complexes *in cis*. This further diversifies the repertoire of self-recognition determinants [39].

*Drosophila* Down's syndrome cell adhesion molecule 1 (Dscam1) provides a classical example of a self-avoidance mechanism in invertebrates [26] (Figure 1A). Dscam1 is a transmembrane protein that consists of an N-terminal ectodomain, a transmembrane segment and a C-terminal tail involved in intracellular signalling [26]. In addition to other elements, the extracellular part of Dscam1 contains three variable immunoglobulin domains, Ig2, Ig3 and Ig7. Their variability is ensured by the complex AS structure of the *Dscam1* gene containing three clusters of mutually exclusive cassette exons. In *Drosophila melanogaster*, this includes 12 variants of exon 4, 48 variants of exon 6 and 33 variants of exon 9 encoding corresponding parts of Ig2, Ig3 and Ig7 (Figure 1A). Selecting one exon from each of these clusters by AS can generate up to 12×48×33=19008 distinct combinations, comparable to the total number of genes in the *D. melanogaster* genome. Isolated Ig2, Ig3 and Ig7 domains can interact homophilically with a surprisingly high specificity [27], thus ensuring strictly homophilic dimerization of full-length Dscam1 ectodomains [28].

Exon 4, exon 6 and exon 9 cluster variants appear to be selected in a stochastic mutually exclusive manner [29]. According to single-cell RT-PCR data, this generates 8–30 distinct Dscam1 splice isoforms co-expressed per neuron at any given time [17,18]. Blending different isoforms in the same neuron endows it with unique surface identity. Indeed, choosing 8 isoforms out of 19008 possibilities at random yields 19008!/(8! × 19000!)=4.22×10^29^ combinations and for 30 isoforms this grows to 19008!/(30! × 18978!)=8.60×10^95^. Both numbers drastically exceed the overall number of neurons in *Drosophila* estimated at ∼1.35×10^5^ [30]. As a result, the likelihood of homophilic interactions between sister branches expressing an identical combination of Dscam1 molecules is substantially higher than between branches of different neurons expressing distinct Dscam1 repertoires.

Homophilic interactions between Dscam1 molecules exposed on different branches (i.e. *in trans*) trigger repulsion and ensure that axonal branches of individual mushroom body neurons and dendritic branches of dendritic arborization neurons spread over sufficiently wide receptive fields without interfering with projections emanating from other neurons [26] (Figure 1A). Consistent with an apparent excess of AS combinations over the number of neurons, genetically modified flies can tolerate a limited loss of the arrayed *Dscam1* exons [31].

Recent work on axon branching in *Drosophila* mechanosensory neurons has identified another reason for co-expressing several Dscam1 variants in the same cell [32,33]. According to the authors’ model, isoform diversity effectively minimizes probability of Dscam1 dimerization within the same plasma membrane plane (i.e. *in cis*). This appears to protect developing axon from excessive activation of a downstream signalling pathway modulated by Slit and the receptor tyrosine phosphatase RPTP69D [32,33]. This function might be specific to mechanosensory neurons since no obvious cell-autonomous Dscam1 effects have been identified in other types of neurons [26].

Molecular mechanisms underlying the choice of a single Dscam1 exon from mutually exclusive possibilities are best described for the exon 6 cluster. In this case, a single upstream docking site base-pairs with a stochastically chosen selector sequence preceding each exon 6 variant [34]. This long-range interaction promotes localized dissociation of the splicing repressor hrp36 followed by recruitment of SR proteins that activate the inclusion of the selected exon-6 unit into mature mRNA [35]. Optimal splicing of this exonic cluster also requires an RNA locus control region preceding the docking site [36].

Vertebrate *Dscam* genes lack extensive AS structure and neuronal self-recognition in this system depends on surface proteins encoded by clustered protocadherin genes. In mice, 3 protocadherin clusters, *Pcdha, Pcdhb* and *Pcdhg*, encode 14, 22 and 22 distinct *Pcdh-α, Pcdh-β* and *Pcdh-γ* surface determinants that can interact homophilically *in trans*, i.e. when expressed on different cell surfaces [37] (Figure 1B). Similar to Dscam1 in *Drosophila*, stochastically generated complements of Pcdh-α, Pcdh-β and Pcdh-γ are thought to mediate self-avoidance of neurites emanating from the same but not distinct neurons [37,38]. The ability of different Pcdh molecules expressed in the same cell to heterodimerize *in cis* provides an additional means for expanding the complexity of the surface code [39,40] (Figure 1B).

*Pcdhbs* are single-exon genes, whereas *Pcdha* and *Pcdhg* clusters contain arrays of first exons encoding the variable extracellular segment followed by three constitutive exons encoding the invariant intracellular domain (Figure 1B). Each first exon is preceded by a promoter that can be stochastically activated through a mechanism involving CTCF- and cohesin-mediated pairing with enhancer elements [37]. Randomly activated first exons are then spliced with the downstream invariant part of the pre-mRNA. Importantly, single neurons often express several alternative Pcdha and Pcdhg isoforms detectable by RT-PCR [19].

## Modulating protein functions

Formation of functional synapses in the vertebrates depends on interaction of presynaptically enriched proteins neurexins (NRXs) with their post-synaptic partners [41,42]. Each of the three genes encoding NRXs in mammals (*Nrxn1*, *Nrxn2* and *Nrxn3*) contains two alternative promoters giving rise to the long (α) and short (β) protein variants. Along with combinatorial splicing at several alternative positions, SS1–SS6 for α and SS4–SS5 for β pre-mRNA, this can generate thousands distinct NRX isoforms [43,44].

Inclusion or skipping of alternative exons is known to modulate interaction of NRXs with its partners including the neuroligin protein family, leucine-rich repeat transmembrane neuronal protein 2 (LRRTM2) and cerebellin (Cbln) [41,42,45,46]. Genetically induced constitutive inclusion of the *Nrxn3* SS4 exon has been shown to reduce recruitment of AMPA receptors to post-synaptic sites possibly as a result of altered trans-synaptic interactions between the NRX3 SS4-included [SS4(+)] isoform and LRRTM2, which is also known to interact with AMPA [47].

*Nrxn* promoters and alternative exons are extensively regulated as a function of neuronal type, maturity and physiological status [41,42]. This might allow different groups of neurons to modulate their synaptic properties by recruiting distinct repertoires of NRX ligands. Recent single-cell RT-qPCR analyses [48] confirmed this conclusion by showing that different types of neurons express characteristic repertoires of Nrxn isoforms. Interestingly, cortical projection neurons express almost exclusively SS4-skipped [SS4(−)] Nrxn1 and Nrxn3 mRNAs, whereas parvalbumin-positive interneurons and D2 receptor-positive medium spiny neurons (MSNs) tend to express a nearly equimolar mixture of the SS4(−) and the SS4(+) Nrxn1 mRNAs. Both D1 and D2 receptor-positive MSNs additionally contain comparable amounts of the SS4(−) and the SS4(+) Nrxn3 isoforms. It is tempting to speculate that isoform co-expression may expand NRX ligand repertoires in specific groups of neurons thus modulating their synaptic connectivity.

**Figure 2:**
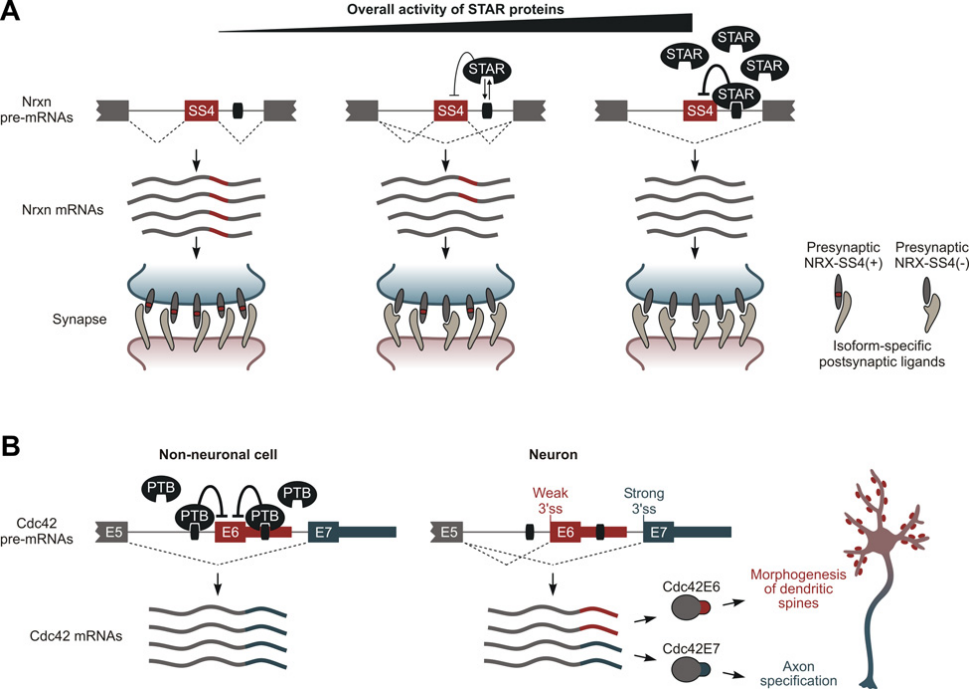
Isoform co-expression can diversify protein functions at a single-cell level (**A**) NRX SS4(+) and SS4(−) isoforms may co-occur in cells with intermediate STAR protein levels. Presynaptic terminals co-expressing a mixture of corresponding NRX variants are expected to interact with a wider range of postsynaptic ligands compared with terminals expressing either of these the two variants individually. (**B**) Co-expression of the Cdc42E6 and the Cdc42E7 isoforms in neurons is regulated by PTB-dependent and constitutive splicing mechanisms, and required for proper development of axons and dendritic spines. See text for details.

The ability of different types of neurons to produce distinct combinations of the SS4(−) and the SS4(+) AS isoforms is controlled by a combination of the STAR family proteins, Sam68/Khdrbs1, SLM-1/Khdrbs2 and SLM-2/T-STAR/Khdrbs3 [49–51] (Figure 2A). Sam68 is expressed ubiquitously in brain, whereas SLM-1 and SLM-2 show largely non-overlapping region- and neuron type-specific expression patterns [50,51]. All three RNA-binding proteins inhibit inclusion of SS4 exons in Nrxn1 and Nrxn3 by interacting with a downstream AU-rich intronic splicing silencer and SLM-1 and SLM-2 additionally promote skipping of SS4 in Nrxn2 [50,51]. Interestingly, Sam68 activity can be stimulated by depolarization-induced phosphorylation at a specific serine residue [49]. SLM-1 and SLM-2 lack this residue and are therefore thought to function in a constitutive manner [50,51]. Thus, co-expression of the SS4(−) and the SS4(+) NRXs might occur when combined activity of the three STAR proteins in the cell is at an intermediate level (Figure 2A).

Our recent study points at wider functional importance of the isoform co-expression scenario [52] (Figure 2B). Using a newly introduced isoform diversity metric we identified a subset of genes that increased co-expression of mRNAs with distinct 3′-terminal exons during neuronal differentiation. One of these genes, *Cdc42*, encodes an important regulator of cell polarity and actin cytoskeleton dynamics and contains two alternative 3′-terminal exons, E7 and E6. E7-terminated mRNAs are expressed ubiquitously, whereas the E6 exon is included selectively in neurons. Notably, single-cell RT-PCR and single molecule RNA FISH analyses suggest that–instead of completely switching from E7 to E6–neurons stably co-express comparable amounts of the two isoforms.

Isoform-specific overexpression and knockdown experiments in primary neurons and evidence from knockout mice lacking E6 and containing increased amounts of the E7-terminated isoform in brain support the model that neurons require Cdc42E7 for axonogenesis and Cdc42E6 for proper development of dendritic spines (Figure 2B). This intracellular segregation of duties may explain, at least in part, the striking variety of neuronal functions of Cdc42 reported in earlier studies [53–57]. Of note, E6 and E7 encode different variants of C-terminal CAAX motifs that may specify cellular localization patterns of the two Cdc42 isoforms through distinct lipid modifications. Prenylation of the E7-encoded CAAX stimulates Cdc42 docking to cellular membranes. On the other hand, the E6-encoded peptide can be both prenylated and palmitoyated, which may target Cdc42E6 to dendritic spines [55,58,59].

Our work also suggests that polypyrimidine tract-binding protein PTB/hnRNP-I//Ptbp1 represses E6 inclusion in non-neuronal cells and that the microRNA *miR-124* likely relieves this repression by dampening PTB levels in neurons [52,60]. Interestingly, balanced utilization of the E6 and E7 exons in neurons depends on the splicing acceptor site of E6 to be relatively weaker than its downstream E7 competitor. This effectively equalizes E6 and E7 odds to be included into mature mRNA in the absence of *trans*-acting splicing repressors (Figure 2B).

## Maintaining gene expression homoeostasis

Negative feedback mechanisms maintaining expression levels of many splicing regulators provide yet another illustration of biological functionality of the isoform co-expression status. These proteins often bias AS of their own pre-mRNA to generate aberrant mRNA products that are typically destabilized by appropriate quality control mechanisms [61,62]. This creates a mutually antagonistic relationship between productive mRNA species increasing the regulator pool and aberrant transcripts that effectively deplete it by diverting pre-mRNA to a non-productive route. In the absence of other regulation inputs, the system is expected to reach a stable equilibrium co-expressing substantial amounts of both productive and non-productive transcripts in the same cell.

**Figure 3:**
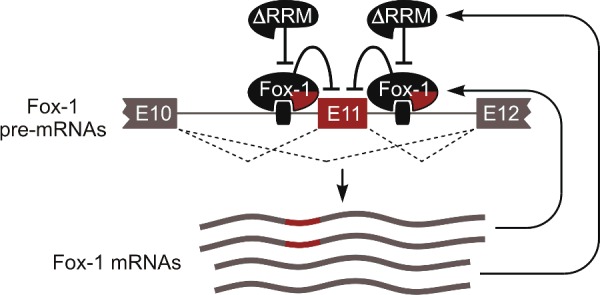
Homoeostatic regulation of Fox-1 abundance through co-expression with its dominant negative isoform. See text for details

Most non-productive mRNAs contain features limiting their half-life, for example, premature stop codons triggering nonsense-mediated decay or retained introns that may promote nuclear degradation [61,62]. However, mammalian Fox-1/A2BP1/Rbfox1 and Fox-2/RBM9/Rbfox2 RNA-binding proteins use a different mechanism to auto-regulate their activity (Figure 3). These proteins repress a conserved 93-nucleotide exon in their own pre-mRNAs by interacting with adjacent intronic motifs mRNAs lacking exon are relatively stable and give rise to readily detectable amounts of dominant negative variants of Fox proteins lacking a part of their RNA-binding domain (RRM) [63]. These shortened ΔRRM protein isoforms have dramatically reduced RNA-binding activity and are thought to antagonize Fox activity by dimerizing with their full-length counterparts or competing for essential co-regulators (Figure 3). Although the above study did not use single-cell detection approaches, this homoeostatic circuitry should favour and, indeed, rely on lasting co-expression of full-length and the ΔRRM isoforms in the same cell.

## Conclusions and future perspectives

We conclude that isoform co-expression in individual cells represents a recurring scenario and predict that ongoing single-cell analyses will identify further examples of such regulation. AS-mediated diversification of cellular transcriptomes and proteomes may afford important functional advantages that are difficult or impossible to gain using other mechanisms. We also note that single-cell co-expression is often an actively regulated state that depends on co-ordinated activity of specific *cis*-regulatory elements and *trans*-acting factors.

An important future challenge in this field will be improving single-cell and single-molecule technologies to an extent where all or at least most RNA molecules expressed in a cell can be detected in an unbiased fashion. It is often assumed that transcriptional profile of a cell is an acceptable proxy for its protein composition. However, translational and post-translational regulation mechanisms may distort this relationship to various degrees depending on the cell type and the isoform identity. It will be interesting to see if rapidly developing proteomics approaches will eventually allow robust detection of protein isoforms with a single-cell resolution.

Revisiting known examples of AS isoform co-expression at the level of tissues and cell types and asking whether the co-expression status holds for individual cells will be another productive direction for future work. Combined with high-throughput approaches, such focused studies will undoubtedly provide new insights into biological functions of AS and mechanisms underlying morphological and functional differences between individual cells.

## Abbreviations

AS: alternative splicing
Dscam1: Down's syndrome cell adhesion molecule 1
LRRTM2: leucine-rich repeat transmembrane neuronal protein 2
MSN: medium spiny neuron
NRX: neurexin
PTB: polypyrimidine tract-binding protein
scRNA-seq: single-cell RNA sequencing
